# Prophylactic Sclerotherapy for Oesophageal Varices

**DOI:** 10.1155/1993/28975

**Published:** 1993

**Authors:** K.-J. Paquet

**Affiliations:** Department of Surgery Heinz-Kalk-Klinik Postfach 2180, Am Gradierbau 3, D-8730 Bad Kissingen Germany


without underlying abdominal pathology. As far as percutaneous drainage is concerned, it certainly is a reliable procedure by which pus and necrotic debris may be removed and the abscess cavity washed. Nevertheless I do not believe that it will be of any help in treating multiple, superficially located abscesses or in the management of patients with severe sepsis and associated pathology such as in acute suppurative cholangitis where the obstruction of the biliary tract results in multiple abscesses scattered throughout both hepatic lobes and severe sepsis occurs accounting for the high mortality rate.

The most correct therapeutic approach to pyogenic liver abscess requires an accurate diagnostic work-up aimed at precisely defining size, location and the number of lesions as well as the type of pathogens involved. In dealing with this desease, one last point deserves to be mentioned, the basic rule from times long past, which still holds good: *Ubi pus ibi evacuat.*

### REFERENCES

1. Ochsner, A., De Bakey, M. and Murray, S. (1938) Pyogenic abscess of the Liver. *Am. J. Surg.*, **40**, 292–314
2. King-Teh Lee, Pai-Ching Sheen, John-Shyong Chen, Chen-Guo Ker (1991) Pyogenic liver abscess: multivariate analysis of risk factors. *World J. Surg.*, **15**, 372–377
3. Branum, G.D., Tyson, G.S., Branum, M.A. and Meyers, W.C. (1990) Hepatic abscess: changes in etiology, diagnosis and management. *Ann. Surg.*, 212, **6**, 655–662
4. Bissada, A.A. and Bateman, J. (1991) Pyogenic liver abscess: a 7-year experience in a large Community Hospital. *Hepato-Gastroent*, **38**, 317–319

Professor D. D'Amico
Universita Degli Studi di Padova
Via Giustianini 2
35128 Padova
Italy

### PROPHYLACTIC SCLEROTHERAPY FOR OESOPHAGEAL VARICES

### ABSTRACT

*The Veterans Affairs Cooperative Variceal Sclerotherapy Group. (1991) Prophylactic sclerotherapy for esophageal varices in men with alcoholic liver disease. The New England Journal of Medicine;* **324**: 1779–1784.

***Background.*** **Sclerotherapy is an effective treatment for bleeding esophageal varices in patients with alcoholic liver disease. It has also been suggested that sclerotherapy might be effective in preventing initial episodes of bleeding and improving survival among such patients.**

***Methods.*** **We conducted a prospective, randomized trial comparing prophylactic sclerotherapy and sham therapy in 281 men with alcoholic liver disease who had at least three variceal channels and no history of variceal bleeding. All the patients underwent endoscopy; 143 received sclerotherapy, and 138 received sham therapy.**

***Results.*** **The two patient groups were well matched at entry with respect to the extent of liver disease and other clinical indexes, except that other medical illnesses were significantly more common in the sclerotherapy group. The study's data-monitoring board terminated the trial 22.5 months after it began because the rate of mortality from all causes was singificantly higher in the sclerotherapy group (32.2 percent) than in the sham-therapy group (17.4 percent, $p = 0.004$), despite the fact that the men who received sclerotherapy had significantly fewer episodes of esophageal variceal bleeding. The causes of death varied, and there is no obvious explanation for the excess mortality in the sclerotherapy group. After the termination of treatment, the excess mortality rate in the sclerotherapy group promptly declined. There were 53 episodes of upper gastrointestinal bleeding (including 10 from esophageal varices and 9 from esophageal ulcers) in the sclerotherapy group and 40 episodes (including 19 from esophageal varices) in the sham-therapy group. Complications of sclerotherapy were frequent but seldom life-threatening.**

***Conclusions.*** **For unknown reasons, prophylactic sclerotherapy is associated with increased mortality among men with moderate-to-severe alcoholic liver disease and esophageal varices. Sclerotherapy should not be performed until after an initial episode of bleeding from esophageal varices has occurred. (N.Engl.J.Med., 1991; 324: 1779–1784.)**

### PAPER DISCUSION

KEY WORDS: Oesophageal varices, sclerotherapy

Endoscopic sclerotherapy is used to prevent rebleeding from varices that have previously bled and to stop hemorrhage from actively bleeding esophageal varices. Elective sclerotherapy in patients who survive the first variceal bleed reduced these patients long-term rebleeding rates and their long-term mortality[1-3]. Emergent sclerotherapy performed within four hours of admission in patients with actively bleeding varices significantly decreases the proportion of patients who continue to bleed twelve hours after admission, and thus, decreases the in-hospital mortality in one study[4]. This study could not be confirmed in a later controlled trial[5]. Unfortunately, variceal bleeding is the initial presentation for 50% of cirrhotic patients[6], and the mortality rate with the first variceal bleed is 30 to 50%[7]. Therefore, more than ten controlled trials have examined the efficacy of sclerotherapy in patients with esophageal varices that never bled[8-20]. These studies of prophylactic sclerotherapy have reached conflicting conclusions. By using meta-analysis to determine the efficacy of prophylactic sclerotherapy including all English-language articles reporting results of randomized controlled trials of prophylactic sclerotherapy in adults, prophylactic sclerotherapy reduced the 13th months mortality rate by 11% (95% confidence interval, 4% - 19%), which represents a 41% relative reduction in mortality rate. Across studies, the mortality rate reductions were positively correlated with the bleeding rate reduction and negatively correlated with complications rate.

On the contrary a recent study of prophylactic sclerotherapy by Gregory *et al.*, (being the representative of the Veterans Affairs Cooperative Variceal Sclerotherapy Groups — thus, this study was exclusively performed in Veterans Administration Hospitals) showed an unexpectedly high mortality rate in the treated group (32.2%, as compared with 17.4% in the control group). Child's score and sclerotherapy were the main predictors of mortality in this study, but the increased mortality in the sclerotherapy group persisted after an adjustment for Child's score and for other medical illnesses. Sclerotherapy was therefore considered an important factor causing excessive mortality. The authors concluded, that prophylactic sclerotherapy is dangerous and should not be performed until after the first episode of variceal bleeding.

I believe that these results are not astonishing and that the reason for the high mortality after sclerotherapy are not unknown, as stated in the conclusion of the abstract. Veteran Administration Hospitals from my personal knowledge are not hospitals with the highest medical and technical standard in the United States. This may also be true for the doctors and endoscopists. This is the first factor that may have influenced the final outcome.

Furthermore this study contains two possible additional conflicting factors. First, more men in the sclerotherapy group than in the control group had medical illnesses, such as chronic obstructive pulmonary disease, diabetes mellitus, cardiac disease and cancer. Second, the initial frequency of the sclerotherapy sessions was high (four treatments in the first month). Major esophageal ulceration is known to be associated with a high frequency of sclerotherapy sessions[2].

Although, sclerotherapy was singled out as an important factor that was associated with increased mortality, for a true understanding of these results, more detailed knowledge about the causes of death is needed. Why were the rates of fatal liver failure and infection higher in the sclerotherapy group? What kinds of infection occurred? Were anti-biotics given at the time of sclerotherapy? What were the ages of the patients who died? Was there any difference in mortality among the men who continued to abuse alcohol and those who were abstinent? Although, there were a few episodes of variceal bleeding in the sclerotherapy group, there were more episodes of other upper gastrointestinal bleeding in this group, and more such episodes were fatal. Was bleeding from esophageal ulcers, which occurred only in the sclerotherapy group, an important cause of death? Was bleeding from stress ulcers, possibly influenced by inadequate sclerotherapy a second important cause? Was bleeding from esophageal varices after sclerotherapy a cause of inadequate experience by the endoscopists, thus influencing the negative outcome of this group?

The study by Gregory *et al.* contains a clear warning that prophylactic sclerotherapy may be not beneficial but instead may be associated with increased mortality, at least in a study population composed of older, actively drinking men with a rather high incidence of non-hepatic medical illness. Whether this conclusion also holds for a study population with a high risk of bleeding but an otherwise reasonable prognosis (CHILD's grade A and B) and no other important medical illness remains in our opinion to be proved. Last but not least, prophylactic endoscopic sclerotherapy has, as described above in the meta-analysis, proved to be useful in the hand of experts.

Recently our group finished a prospective controlled randomized trial with a strict selection of patients (20% of all patients with esophageal varices) according to the following factors at risk to bleed: Esophageal varices degrees III and IV (according to our classification)[8], minivarices on top of the varices (so called "cherry red spots") and an elevated portal pressure, measured by wedged occluded hepatic or esophageal variceal pressure over 22mmHg. In this study more than one hundred patients were included. Over a period of three years it could be demonstrated, that prophylactic endoscopic sclerotherapy in a selected group of patients with a high risk of bleeding and performed by doctors with endoscopic expertise is not only able to reduce the frequency of bleeding but also to prolong survival.

Professor K-J Paquet
Department of Surgery
Heinz-Kalk-Klinik
Postfach 2180
Am Gradierbau 3
D-8730 Bad Kissingen
Germany

### REFERENCES

1. Westaby, D., MacDougall, B.R.D. and Williams, R. (1985) Improved survival following injection sclerotherapy for esophageal varices: final analysis of a controlled trial. *Hepatology*, **5**, 827–830
2. The Copenhagen Esophageal Variceal Project (1984) Sclerotherapy after first variceal hemorrhage in cirrhosis: a randomized multicenter trial. *New Engl.J. of Med.* **311**, 1594–1600
3. Infante-Rivard, C., Esnaola, S. and Villeneuve, J-P. (1989) Role of endoscopic variceal sclerotherapy in the long-term management of variceal bleeding. *Gastroenterology*, **96**, 1087–1092
4. Paquet, K-J. and Feussner, H. (1985) Endoscopic sclerosis and esophageal balloon tamponade in acute hemorrhage from esophagogastric varices: a prospective controlled randomized trial. *Hepatology*, **5**, 580--583
5. Westaby, D., Hayes, P.C., Gimson, A.E.S., Polson, R.J. and Williams, R. (1989) Controlled clinical trial of sclerotherapy for active variceal bleeding. *Hepatology*, **9**, 274–277
6. Hayes, P.C., Westaby, D. and Williams, R. (1988) Prophylactic injection sclerotherapy for esophageal varices: a critical approach. *Gastrointestinal Endoscopy*, **34**, 359–361
7. Burrhoughs, A.K., D'Heygere, F. and McIntyre, N. (1986) Pitfalls in studies of prophylactic therapy for variceal bleeding in cirrhotic. *Hepatology*, **6**, 1407–1413
8. Paquet, K-J. (1982) Prophylactic endoscopic sclerosing treatment of the esophageal wall in varices — a prospective controlled randomized trial. *Endoscopy*, **14**, 3–5
9. Paquet, K-J. and Koussouris, P. (1986) Is there an indication for prophylactic endoscopic paravariceal injection sclerotherapy in patients with liver cirrhosis and portal hypertension? *Endoscopy*, **18**, 32–35
10. Witzel, L., Wollbergs, E. and Merki, H. (1985) Prophylactic endoscopic sclerotherapy of esophageal varices: a prospective controlled randomized study. *Lancet*, **I**, 773–775
11. Koch, H., Henning, H., Grimm, H. and Soehendra, M. (1986) Prophylactic sclerosing of esophageal varices — results of a prospective controlled study. *Endoscopy*, **18**, 40–43
12. Santangelo, W.C., Doeno, M.J., Estes, B.L. and Krejs, G. (1988) Prophylactic sclerotherapy of large esophageal varices. *New Engl.J.Med.*, **318**, 814–818
13. Sauerbruch, T., Wotzka, R., Köpcke, W. *et al.* (1988) Prophylactic sclerotherapy before the first episode of variceal hemorrhage in patients with cirrhosis. *New Engl.J.Med.*, **319**, 8–15
14. Piai, G., Cipoletta, L., Claar, M. *et al.* (1988) Prophylactic sclerotherapy of high risk esophageal varices: results of a multicentric prospective controlled trial. *Hepatology*, **8**, 1495–1500
15. Russo, A., Giannone, G., Magnano, A., Passanisi, G. and Lungo, C. (1989) Prophylactic sclerotherapy in non-alcoholic liver cirrhosis: preliminary results of a prospective randomized trial. *World J.Surg.*, **13**, 149–153
16. Pötzi, R., Bauer, P., Reichel, W., Kerstan, E., Renner, F. and Gangl, A. (1989) Prophylactic endoscopic sclerotherapy of esophageal varices in liver cirrhosis. A multicenter prospective controlled randomized trial in Vienna. *Gut*, **30**, 873–879

17. Kobe, E., Zipprich, P., Schentke, K-U. and Nilius, R. (1990) Prophylactic endoscopic sclerotherapy of esophageal varices — a prospective controlled randomized trial. *Endoscopy*, **22**, 245–248
18. The Veterans Affairs Cooperative Variceal Sclerotherapy Group (1991) Prophylactic sclerotherapy for esophageal varices in men with alcoholic liver disease: a randomized, single blind, multicenter clinical trial. *New Engl.J.Med.*, **324**, 1779–1784
19. Trigger, D.R., Smart, H.L., Hoskin, S.H.W. and Johnston, A.L. (1991) Prophylactic sclerotherapy for esophageal varices: long-term results of a single center trial. *Hepatology*, **13**, 117–123
20. de Franchis, R., North Italian Endoscopic Club (1991) Prophylactic sclerotherapy in high risk cirrhotics selected by endoscopic criteria — a multicenter randomized controlled trial. *Gastroenterology*, **101**, 1087–1093

### BUDD-CHIARI SYNDROME - TRANSPLANT, MESO-ATRIAL SHUNT OR COMBINED PORTOCAVAL SHUNT WITH CAVO-ATRIAL SHUNT

### ABSTRACT

*Orloff, M.J. and Daily, P.O. (1992) Treatment of Budd-Chiari syndrome due to inferior vena cava occlusion by combined portal and vena caval decompression. The American Journal of Surgery; 163: 137–143.*

**This study concerns Budd-Chiari syndrome (BCS) caused by occlusion of the subdiaphragmatic inferior vena cava (IVC). It describes the experimental and clinical evaluation of the treatment of this disorder by one-stage combined portal and vena caval decompression with a direct side-to-side portacaval shunt (PCS) and a caval-atrial shunt (CAS) graft. BCS was produced in rats by gradual occlusion of the suprahepatic IVC with an ameroid constrictor. When ascites and portal hypertension were established, 12 control rats survived a sham thoracolaparotomy, 16 rats survived a mesoatrial shunt, and 16 rats survived combined PCS and CAS graft. All control rats re-formed ascites and died within 2 months. Nine of 16 rats with mesoatrial shunt developed graft thrombosis, re-formed ascites, and died within 2 months. In contrast, only 2 of 16 rats that underwent combined PCS and CAS developed graft thrombosis, re-formed ascites, and died. Liver biopsies showed reversal of severe pathologic changes in rats with patent grafts. Clinical evaluation of combined PCS and CAS using a 20-mm ring-reinforced Gore-Tex graft has been undertaken in five patients with BCS and ascites, hepatosplenomegaly, intense hepatic congestion on biopsy, and angiography showing occlusion of both the IVC and hepatic veins. All five patients were alive and well 6 months to 7.5 years postoperatively with patent grafts, no ascites or need for diuretics, no encephalopathy, normal liver function, and reversal of liver pathology. It is concluded that combined PCS and CAS create a high-flow shunt that decompresses both the portal system and IVC, has a low incidence of graft thrombosis, has been consistently effective in relieving BCS caused by IVC occlusion, and appears to be superior to mesoatrial shunt.**

